# Effects of Dietary Aflatoxin B1 on Hybrid Grouper (*Epinephelus fuscoguttatus* ♀ × *Epinephelus lanceolatus* ♂) Growth, Intestinal Health, and Muscle Quality

**DOI:** 10.1155/2024/3920254

**Published:** 2024-02-20

**Authors:** Hao Liu, Ruitao Xie, Weibin Huang, Yuanzhi Yang, Menglong Zhou, Baiquan Lu, Biao Li, Beiping Tan, Xiaohui Dong

**Affiliations:** ^1^Laboratory of Aquatic Animal Nutrition and Feed, College of Fisheries, Guangdong Ocean University, Zhanjiang 524088, China; ^2^Aquatic Animals Precision Nutrition and High-Efficiency Feed Engineering Research Centre of Guangdong Province, Zhanjiang 524088, China; ^3^Key Laboratory of Aquatic, Livestock and Poultry Feed Science and Technology in South China, Ministry of Agriculture and Rural Affairs, Zhanjiang 524000, China; ^4^Guangdong Evergreen Feed Industry Co., Ltd., Zhanjiang 524000, China

## Abstract

This study investigated the effects of varying doses of dietary aflatoxin B1 (AFB1) on the growth, intestinal health, and muscle quality of hybrid grouper. Four diets with varying AFB1 concentrations (0, 30, 445, and 2,230 *μ*g kg^−1^) were used. Elevating AFB1 concentrations led to a decline in growth indexes, specifically the weight gain rate and the specific growth rate, although the survival rate remained unchanged. Morphological indicators showed a dose-dependent decline with AFB1 exposure. Intestinal MDA content and hindgut reactive oxygen species (ROS) levels increased, while antioxidant indexes and digestive enzymes decreased with higher AFB1 levels. AFB1 negatively influenced hindgut tight junction protein and antioxidant-related gene expression while promoting inflammation-related gene expression. The presence of AFB1 in the experiment led to a decrease in beneficial intestinal bacteria, such as *Prevotella*, and an increase in harmful intestinal bacteria, such as *Prevotellaceae_NK3B31_group*. Muscle lipid and unsaturated fatty acid content significantly decreased, while muscle protein and liver AFB1 content increased dramatically with higher AFB1 concentrations. AFB1 caused myofibrillar cleavage and myofilament damage, leading to increased spaces between muscle fibers. In conclusion, diets with AFB1 levels exceeding 30 *μ*g kg^−1^ inhibited hybrid grouper growth, while levels surpassing 445 *μ*g kg^−1^ resulted in hindgut ROS accumulation, inflammation, elevated intestinal permeability, reduced digestive enzyme activity, and compromised muscle quality.

## 1. Introduction

The concurrent growth of population and income in developing nations has resulted in a concomitant escalation in the demand for and purchasing power of aquatic products. This surge in demand has been effectively addressed by the rapid expansion of aquaculture [1]. According to the Food and Agriculture Organization (FAO) [1], there was a notable rise in per capita seafood consumption from 1961 to 2015. Specifically, the average amount of seafood consumed per person grew from 9 kg in 1961 to 20 kg in 2015. According to the FAO [1], in 2020, the aquaculture industry experienced a notable growth of 5.8% per annum from 2000 to 2010, surpassing the growth rates of all other sectors involved in meat production. Furthermore, this growth persisted at 4.5% annually from 2010 to 2018. The exponential growth of aquaculture has facilitated the widespread availability of aquatic items to the general populace. Nevertheless, using plant protein as a substitute for fishmeal in order to address the scarcity issue is not without its drawbacks, one of which is the potential risk of mycotoxin contamination [2]. Despite the use of many protective measures, mycotoxin contamination in the dietary intake remains nearly unavoidable. Furthermore, it has been observed that the deleterious consequences resulting from mycotoxins can be mitigated to some extent, but complete eradication has been touted as unachievable [3]. Therefore, further research into the fundamental mechanisms that contribute to the adverse effects of mycotoxins on the growth and well-being of fish is essential. These inquiries will provide fresh perspectives and serve as important benchmarks for subsequent research concerning the contamination of fish diets with mycotoxins.

Secondary metabolites known as aflatoxins are synthesized by two widely distributed fungi, namely *Aspergillus flavus* and *Aspergillus parasiticus*. These fungi are well-known for their ability to contaminate animal feed, posing a significant threat to animal health [4].

Aflatoxin is essential in the pathophysiology of the gastrointestinal system. Chen et al. [5] established a correlation between this condition and the incidence of intestinal damage, which is defined by the death of cells in the lining of the intestines. Additionally, it has been linked to the immunological problems, impaired intestinal function, and decreased efficiency in feed utilization. Prior research has shown that aflatoxin can cause gastrointestinal injury, resulting in a breakdown of the intestinal barrier function. This breakdown leads to intestinal leakage and displacement of microorganisms [6, 7]. The intestinal microbiota has a crucial impact on the host's overall health by performing various functions, including aiding in the digestion and absorption of food, regulating immune responses, participating in bio antagonism, and antiaging processes, and influencing neurobehavioral regulation [8–10]. Hence, it is imperative to preserve the proper physiological functioning of the host gut to promote overall health and facilitate growth. The investigation of the impact of aflatoxin B1 (AFB1) on alterations in intestinal microflora has gained significant attention in the field of determining host health, owing to the vulnerability of intestinal microflora to external stressors [10]. Although, previous studies have examined the toxicity of aflatoxins on various animal species [11–13], our knowledge regarding the precise mechanisms by which AFB1 affects the intestinal microbiota in aquatic animals is still inadequate. Hence, further investigation is necessary to further our understanding in this domain.

Consumers have higher expectations for food nutrition, quality, and safety due to the abundance of material goods, and they expect the flavor quality of farmed grouper to meet the standards of wild grouper. Leeman et al. [14] found that the transfer of mycotoxins from feed to cattle can lead to the buildup of these poisons in the animals. This can pose a direct threat to human health. The accumulation of mycotoxins in land-dwelling animals has been thoroughly examined, and the European Food Safety Authority (EFSA) is currently assessing the potential harm to humans caused by several mycotoxins (Aflatoxin, Ochratoxin A, Zearalenone, Deoxynivalenol, Fumonisins, Tricothecenes−2 toxin, and HT−2 toxin) [15]. Regarding bioconcentration, however, mycotoxins in aquaculture animals have not been extensively studied regarding its bioconcentration.

The hybrid grouper is a crossbreed resulting from the mating of a male giant grouper (*Epinephelus lanceolatus*) and a female brown-marbled grouper (*Epinephelus fuscoguttatus*). The hybrid grouper has gained significant popularity in Southeast Asia and China due to its rapid growth rate, delectable meat, and exceptional nutritional content. Based on the data from the Fisheries Yearbook 2022, China's grouper production reached ∼204,100 tons in 2021. Thus yet, no research pertaining to AFB1 and its effects on groupers has been observed. Groupers cannot directly benefit from AFB1 studies conducted on other species due to interspecific variations. Hence, it is crucial to investigate the impact of dietary AFB1 on the growth, intestinal health, accumulation in different tissues, and muscle quality of grouper.

## 2. Materials and Methods

### 2.1. Animal Ethics Statement

The animal experiments were conducted in strict adherence to the guidelines outlined in the “Guide for the Care and Use of Laboratory Animals” as recommended by the National Institutes of Health. The Animal Ethics Committee of Guangdong Ocean University accepted the animal protocols (approval ID: GDOU-IACUC−2022-A0502; approval date: 2nd May 2022).

### 2.2. Experimental Diets Preparation

Table 1 displays the constituent elements of the basal diet. The diet included brown fish meal, soybean protein concentrate, wheat gluten, and chicken meal as sources of dietary protein. The dietary lipid sources utilized were fish oil and soybean lecithin. The purified AFB1, with a purity level over 99%, was acquired from Pribolab (Pribolab Pte. Ltd. Singapore). Initially, AFB1 was dissolved in 2 mL of chloroform that was warmed. Subsequently, the heated chloroform was combined with 50 mL of fish oil after complete dissolution. Subsequently, the chloroform was completely volatilized in a fume hood. Then, toxic fish oil, concentrated in grades of 50, 500, and 2,500 *μ*g kg^−1^ (measured values 30, 445, and 2,230 *μ*g kg^−1^), were mixed with fresh fish oil and added to the diets. The operational parameters for diet preparation were adhered to [16]. The formulated diets were stored at a temperature of −20°C. The paper utilized the ARRIVE reporting criteria [17]. Two distinct scientists, namely Liu Hao, were assigned to weigh the diet for each animal. The treatment group assignment was known exclusively by this investigator. Zhou Menglong, the second investigator, had the responsibility of providing food. The study inquiry was conducted in a manner where the group assignments were kept undisclosed.

### 2.3. Feeding Trial and Sample Collection

The feeding and management in this study adhered to the protocol established by the Agricultural Animal Care Advisory Committee of Guangdong Ocean University. Experiments were carried out in an indoor marine culture system at Zhanjiang Evergreen South Marine Science & Technology Co., Ltd. The juvenile groupers were acquired from the HongYun fishery located in Zhanjiang, Guangdong, China. Prior to commencing the experiment, the grouper had a 2-week acclimation period in the experimental environment, as stated by Liu et al. [18]. Subsequently, a total of 360 groupers with an average weight of 11.59 ± 0.03 g were randomly distributed among 12 fiberglass buckets, each with a capacity of 500 L. The buckets were divided into four treatment groups, with each treatment being replicated three times and including 30 fish each bucket. For 56 days, fish were fed the experimental diet twice daily (8:00–9:30 a.m. and 4:00–5:30 p.m.) until they appeared satiated. The unconsumed diet was gathered following a 30-min period of feeding [18], dehydrated, and measured to ascertain the amount consumed. The pH and water temperature were monitored during the experiment, with values of 7.4 ± 0.3 and 29.5 ± 2.0°C, respectively. Additionally, the dissolved oxygen levels were found to be above 6.0 mg L^−1^. The experiments were conducted throughout diurnal cycles.

Following the conclusion of the experiment, the fish contained within each fiberglass bucket were deprived of diet for a period of 24 hr. After anesthetics (1,3-Dimethoxy-2-hydroxybenzene, 100 mgL^−1^, Aladdin, Shanghai, China) were used, the fish were weighed and counted, growth-related indices were calculated, and then the fish that needed to be sampled were euthanized by manual stunning. Three fish were randomly chosen from each bucket and promptly weighed, while their body length was measured to compute the morphological index using the methodology established by Liu et al. [18]. Then, 12 more fish were picked at random from each bucket. Gut and muscle were put in enzyme-free drum tubes, frozen in liquid nitrogen, and kept at −80°C so that the enzyme activity, microflora, and reactive oxygen species (ROS) levels of the gut and muscle could be measured in real time. The muscles and guts of three randomly chosen fish were washed with saline and then put in a 4% paraformaldehyde solution right away so that they could be studied histopathologically. After the gut was taken, muscle from the rest of the fish was put in a bag and sealed so that amino acids, fatty acids, and muscle proximate analysis could be done. To test the amounts of AFB1 in tissues, leftover liver, and muscle were put in sealed bags.

### 2.4. Sample Preparation and Biochemical Analysis

The Association of Official Analytical Chemists [19] established methods for determining dry matter (105°C), crude protein (by Kjeldahl apparatus, nitrogen × 6.25), and crude lipid (extraction with petroleum ether by Soxhlet apparatus) in raw materials, diets, and muscle. AFB1 levels in the liver and muscles were measured using the Pribolab AFB1 ELISA kit (Pribolab Pte. Ltd. Singapore). The amount of AFB1 in the diet was determined using the agriculture industry standard of the People's Republic of China: NY/T2071-2011.The activity of intestinal enzymes and antioxidant parameters (MDA: malondialdehyde, SOD: superoxide, CAT: catalase, ROS, T-AOC: total anti-oxidation capacity, GPx: glutathione peroxidase, LIP: Lipase, AMS: *α*-amylase, TRY: Trysin) were assessed following the directions provided by the ELISA kit (Shanghai Enzyme-linked Biotechnology Co., Ltd.).

### 2.5. Hindgut and Muscle Histological Observation

Histological analyses were conducted using the methodology outlined in prior research [20]. The hindgut and muscle samples were immersed in Bouin's solution for 24 hr to fix them, and then preserved in ethanol with a concentration of 700 g kg^−1^. Following the removal of water through a process of gradual exposure to different concentrations of ethanol, the samples were then enclosed within paraffin wax. The tissue samples were cut into sagittal slices that were 5–7 *μ*m thick. These sections were then stained with hematoxylin–eosin (H&E) and made ready for examination using a Nikon ECLIPSE 80i microscope from Nikon Corporation in Kanagawa, Japan.

### 2.6. Hindgut ROS Assay

Tissue ROS quantification is a technical method that employs a transmembrane fluorescent stain, DCFH-DA (2′, 7′-Dichlorodihydrofluorescein diacetate), to quantify the presence of ROS in cryopreserved tissues. Upon oxidative injury to tissues, the stain fluoresces. The procedure was executed in accordance with the guidelines supplied by the Wuhan Servicebio Biotechnology Co., Ltd.

### 2.7. Analysis of the Whole-Intestinal Microbiota

Following the manufacturer's directions, the TGuide S96 Magnetic Soil/Stool DNA Kit (Tiangen Biotech (Beijing) Co., Ltd.) was used to get total genomic DNA from intestine samples [20]. The area V3–V4 of the bacterial 16S rRNA gene that changes a lot was amplified using primer pairs 338 F: 5′-ACTCCTACGGGAGGCAGCA−3′ and 806R: 5′-GGACTACHVGGGTWTCTAAT−3′. We used an Omega DNA purification kit (Omega Inc., Norcross, GA, USA) to clean up the amplified products and Qsep-400 (BiOptic, Inc., New Taipei City, Taiwan, ROC) to measure how much DNA was made. A machine called Illumina novaseq6000 (Beijing Biomarker Technologies Co., Ltd., Beijing, China) was used to pair-end sequence the amplicon library (2× 250). Bioinformatic analysis was done on BMKCloud, which is an online tool for data analysis that is dynamic, real-time, and interactive (https://www.biocloud.net).

### 2.8. Muscle Amino Acid and Fatty Acid Determination

Determination of amino acids utilizing Chinese standard protocols (GB/T18246-2019). By acid-catalyzed transmethylation of total lipids with boron trifluoride–methanol, fatty acid methyl esters were produced and analyzed by gas chromatograph (7890A, Agilent Technologies Inc., US).

### 2.9. Real-Time Polymerase Chain Reaction (PCR) Analysis

RNA isolation, reverse transcription, and quantitative real-time PCR were similar to another lab work [16]. According to the manufacturer's directions, TransZol UP (TransGen Biotech, Beijing, China) collected hindgut and muscle total RNA. As indicated in Liu et al. [16], RNA quality was spectrophotometrically (A260 : 280 nm ratio) and quantity was assessed by agarose gel (1%) electrophoresis. RT was conducted using the Evo M-MLV reverse transcription kit (Accurate Biology, AG11705) per manufacturer's instructions. Using transcriptome and grouper (*E. lanceolatus*) genome sequences, real-time fluorescence quantitative PCR following design-specific primers (Table 2). The gene-specific standard curves from tenfold serial dilutions were used to calculate target and housekeeping gene amplification efficiencies. According to Livak and Schmittgen [21], mRNA expression was evaluated using 2^−*ΔΔ*CT^ after checking primer amplification efficiency of roughly 100%.

### 2.10. Calculation Formula and Statistical Analysis

The subsequent indices and parameters were computed utilizing a standard formula [22, 23]: morphology indices, including condition factor (CF), carcass ratio (CR), and filet yield (FY); feed utilization indices, including feed conversion ratio (FCR); and growth performance parameters, including survival rate (SR), weight gain rate (WGR), and specific growth rate (SGR). Normality and homology analyses were conducted prior to the application of ANOVA. In SPSS version 19 (SPSS, Michigan Avenue, Chicago, IL, USA), the significance of mean differences between treatment groups was ascertained utilizing Tukey's multiple range test. Probability significance tests were conducted at a level of *P* < 0.05. The mean ± SEM (standard error of the mean) is used to represent the data (*n* = 3).

## 3. Results

### 3.1. Growth and Feed Utilization

Table 3 displays the impact of the test diets on the growth and feed consumption of grouper. The SR of different diets ranged from 90.00%−95.56%, without any statistically significant disparities (*P* > 0.05). There was a negative link between the increase in dietary AFB1 level and the values of WGR, SGR, CF, CR, and FY. Group A2230 had the lowest values for all of these parameters (*P* < 0.05). On the contrary, FCR showed a dose-dependent increase of dietary AFB1 and reached the minimum value in group A0 (*P* < 0.05).

### 3.2. Hindgut and Muscle Histopathology

**Figure 1:**
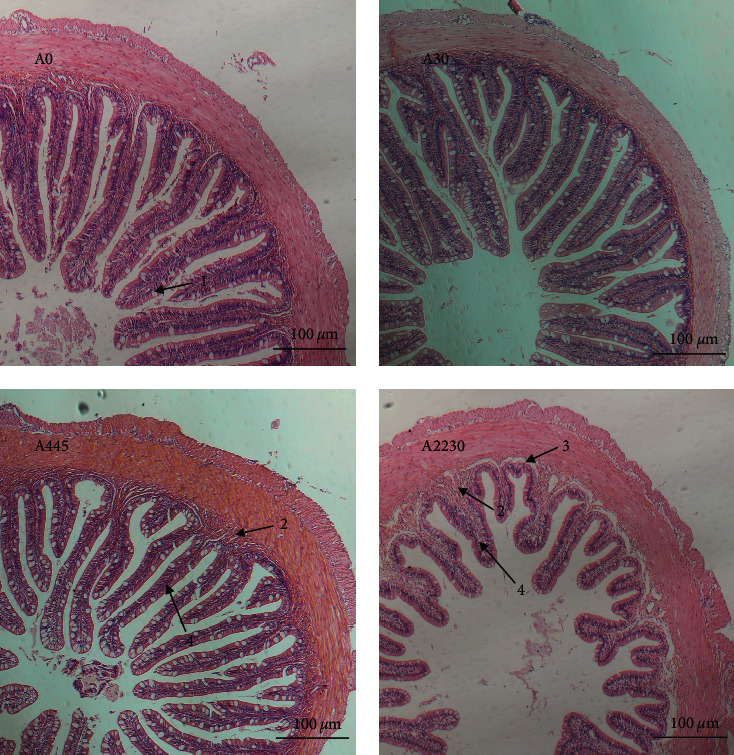
Histopathology of grouper hindgut in control and following a dose-increasing AFB1 exposure. A0 : 0 *μ*g kg^−1^ AFB1, control; A30 : 30 *μ*g kg^−1^ AFB1; A445 : 445 *μ*g kg^−1^ AFB1; A2230 : 2,230 *μ*g kg^−1^ AFB1. 1. Cupped cells; 2. Submucosal lymphocytes; 3. Submucosal separation from muscular layer; 4. Villi. Magnification 200x.

Figure 1 shows micrographs of H&E-stained hindgut cross-sections of grouper under light microscopy. In the medium AFB1 group, there was an observed thickening of the submucosa and a reduction in the width of the villi, as depicted in Figure 1(c), when compared to the control group shown in Figure 1(a). In the group exposed to high levels of AFB1 (Figure 1(d)), the villi were observed to be shorter, the number of cupped cells was decreased, and the submucosa seemed thicker and more detached from the muscle layer, as compared to the control group (Figure 1(a)).

**Figure 2:**
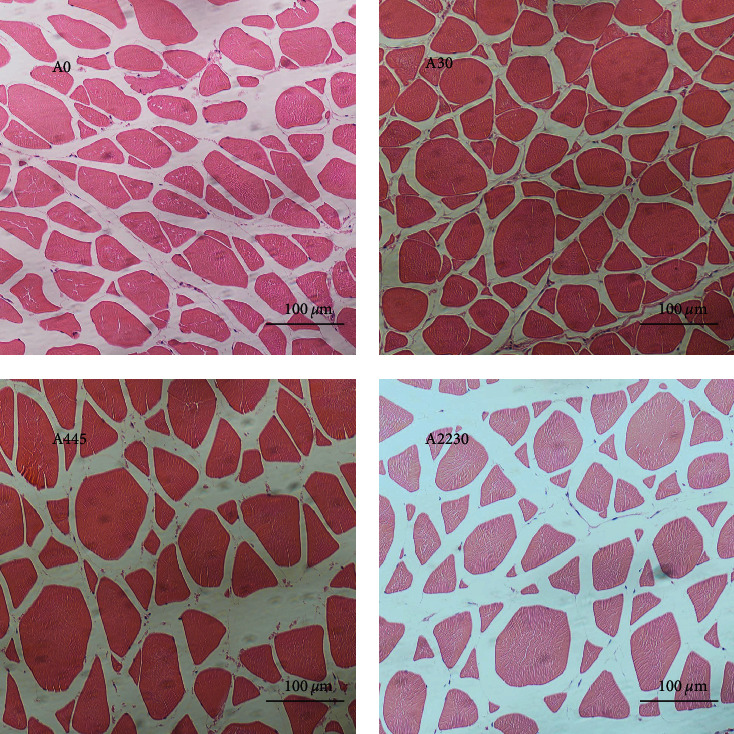
Histopathology of grouper muscle in control and following a dose-increasing AFB1 exposure. A0 : 0 *μ*g kg^−1^ AFB1, control; A30 : 30 *μ*g kg^−1^ AFB1; A445 : 445 *μ*g kg^−1^ AFB1; A2230 : 2,230 *μ*g kg^−1^ AFB1. Magnifcation 200x.

The histopathological changes of grouper muscles after AFB1 exposure are shown in Figure 2. The myogenic fiber interstices and muscle segment lengths exhibited a consistent and organized pattern, characterized by a high density and compact organization, in both the control and 30 *μ*g kg^−1^ AFB1-exposed groups. At 445 *μ*g kg^−1^ AFB1 exposure, the myogenic fibers of grouper showed partial damage, but the texture remained clear. When the AFB1 dose was 2,230 *μ*g kg^−1^, the myogenic fiber cracks gradually increased, producing significant interfibrillar gaps.

### 3.3. Hindgut ROS Fluorescence Staining

**Figure 3:**
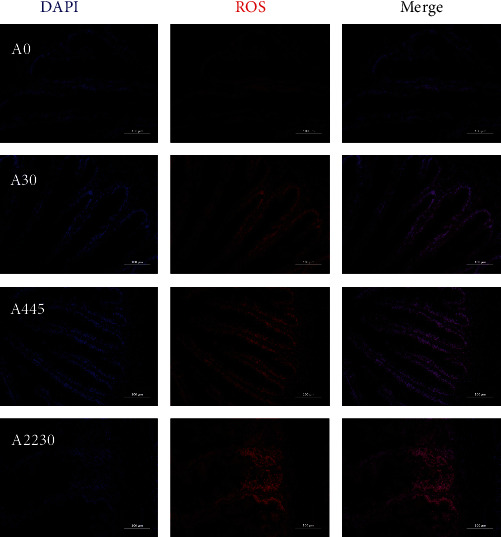
Effect of dietary AFB1 level on liver ROS accumulation in grouper. A0 : 0 *μ*g kg^−1^ AFB1, control; A30 : 30 *μ*g kg^−1^ AFB1; A445 : 445 *μ*g kg^−1^ AFB1; A2230 : 2,230 *μ*g kg^−1^ AFB1.

Hindgut ROS fluorescence staining is shown in Figure 3. The ROS fluorescence intensity in groups A445 and A2230 exhibited a considerably higher magnitude compared to the control group.

### 3.4. Whole-Intestinal Enzyme Activity and Antioxidant Parameters

Table 4 displays the findings of whole-intestinal enzyme activity and antioxidant measures. The investigation revealed that the MDA of group A2230 was much greater than that of group A0 (*P* < 0.05). Additionally, the ROS levels of group A445 and A2230 were also significantly higher than that of group A0 (*P* < 0.05). The levels of antioxidant parameters (SOD, CAT, GPx, T-AOC) in group A2230, which received a high dose of AFB1, were considerably lower than those in group A0 (*P* < 0.05). The levels of intestinal digesting enzymes (LIP, AMS, and TRY) decreased as the dietary AFB1 level increased (*P* < 0.05).

### 3.5. Hindgut and Muscle Gene Expression

**Figure 4:**
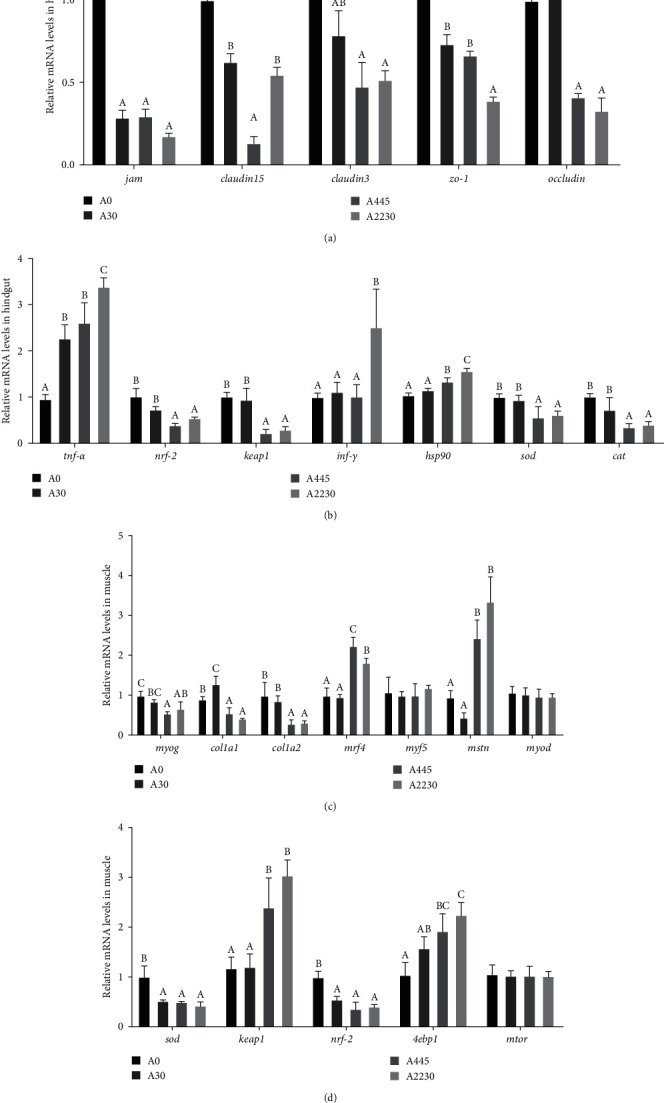
Effect of dietary AFB1 levels on gene expression levels associated with grouper hindgut and muscle. (a) Hindgut tight-junction protein-related gene expression levels. (b) Hindgut antioxidant-related gene expression levels. (c) Muscle production-related genes expression levels. (d) Muscle antioxidant and protein production related genes expression levels. A0 : 0 *μ*g kg^−1^ AFB1, control; A30 : 30 *μ*g kg^−1^ AFB1; A445 : 445 *μ*g kg^−1^ AFB1; A2230 : 2,230 *μ*g kg^−1^ AFB1. *jam*, junctional adhesion molecule 1, *zo-1*: tight junction proteins ZO-1, *tnf-α*: tumor necrosis factor-*α*, *nrf-2*: nuclear factor erythroid 2-related factor 2, *keap1*: kelch-like ECH-associated protein 1, *inf-γ*: interferon-*γ*, *hsp90*: heat shock protein 90, *sod*: superoxide dismutase, *cat*: catalase, *myog*: myogenin, *col1a1*: collagen *α*-1(I) chain, *col1a2*: collagen *α*-2(I) chain, *mrf4*: myogenic factor 4, *myf5*: myogenic factor 5, *mstn*: myostatin, *myod*: myogenic differentiation antigen, *4ebp1*: eukaryotic translation initiation factor 4E binding protein 1, *mtor*: mammalian target of rapamycin. Columns represent the mean ± SEM (*n* = 3). One-way analysis of variance (ANOVA) was used to compare the means. For each index, bars without sharing a common letter indicate significant differences (*P* < 0.05).

The gene expression results are shown in Figure 4

#### 3.5.1. Hindgut Tight-Junction Protein-Related Gene Expression Levels

The gene expression levels of tight junction protein-related genes, such as junctional adhesion molecule 1 (*jam*), *occludin*, *claudin15*, *claudin3*, and tight junction proteins ZO-1 (*zo-1*)) were significantly lower (*P* < 0.05) in both the AFB1 medium-dose group (A445) and the AFB1 high-dose group (A2230) than in the control group (A0).

#### 3.5.2. Hindgut Antioxidant-Related Gene Expression Levels

The expression levels of kelch-like protein 19 (*keap1*), NFE2-like bZIP transcription factor 2 (*nrf-2*), superoxide dismutase (*sod*), and catalase (*cat*) decreased considerably as the dietary AFB1 levels increased (*P* < 0.05). The expression levels of tumor necrosis factor *α* (*tnf-α*), interferon-gamma (*inf-γ*), and heat shock protein 90 (*hsp90*) were markedly elevated in the high-dose group (group A2230) compared to the control group (group A0) in this experiment (*P* < 0.05).

#### 3.5.3. Muscle Production-Related Genes Expression Levels

In this experiment, the expression levels of myogenin (*myog*), *col1a1* (collagen *α*-1 (I) chain), and *col1a2* (collagen *α*-2 (I) chain) were significantly lower (*P* < 0.05) in the medium and high-dose groups (groups A445 and A2230) compared to the control group (group A0). On the other hand, myogenic factor 4 (*mrf4*) and myostatin (*mstn*) were significantly higher (*P* < 0.05) in the medium and high-dose groups (groups A445 and A2230) compared to the control group (group A0). The levels of myogenic factor 5 (*myf5*) and myogenic differentiation antigen (*myod*) did not show significant differences (*P* > 0.05) between the groups.

#### 3.5.4. Muscle Antioxidant and Protein Production-Related Genes Expression Levels

The experiment showed that the levels of *sod* and *nrf-2* expression were considerably greater in the control group (group A0) compared to the treated groups (groups A30, A445, and A2230) (*P* < 0.05). The expression levels of *keap1* and *4 ebp1* showed a substantial rise when the levels of diet AFB1 increased. The greatest expression levels were observed in group A2230 (*P* < 0.05). The expression levels of mTOR did not show any significant differences between the groups (*P* > 0.05).

### 3.6. Changes of Whole-Intestinal Microflora

**Figure 5:**
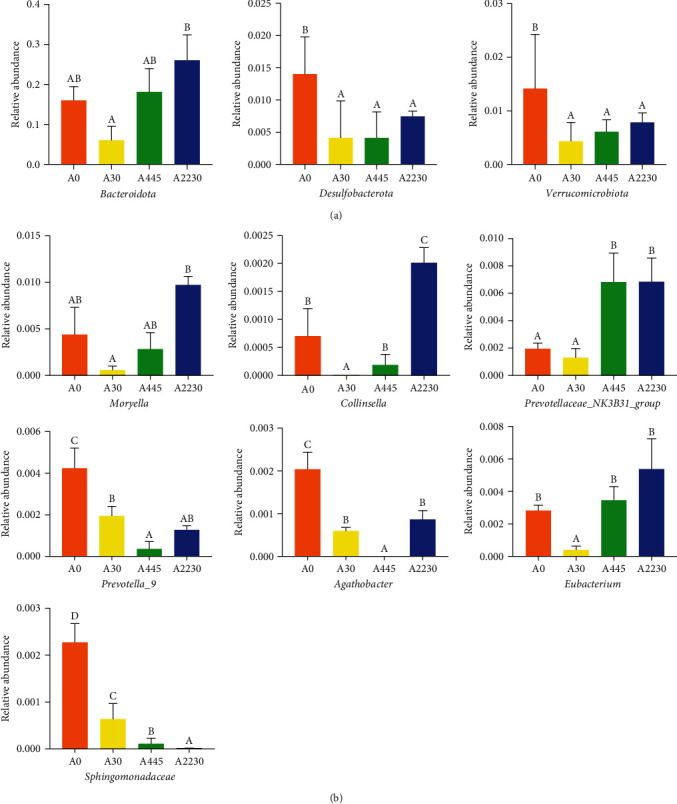
Effect of dietary AFB1 levels on whole-intestinal microflora of groupers. (a) Significant differences in intestinal microflora at the phylum level. (b) Significant differences in intestinal microflora at the genus level. Columns represent the mean ± SEM (*n* = 3). One-way analysis of variance (ANOVA) was used to compare the means. For each index, bars without sharing a common letter indicate significant differences (*P* < 0.05). A0 : 0 *μ*g kg^−1^ AFB1, control; A30 : 30 *μ*g kg^−1^ AFB1; A445 : 445 *μ*g kg^−1^ AFB1; A2230 : 2,230 *μ*g kg^−1^ AFB1.

The results of whole-intestinal microflora are shown in Figure 5.

At the phylum level, the abundance of bacteroidota exhibited a pattern of decline followed by an increase, with the maximum abundance seen in group A2230 (*P* < 0.05). The number of Desulfobacterota and Verrucomicrobiota was substantially greater in the control group compared to the groups exposed to AFB1 (*P* < 0.05). At the genus level, the abundance of *Moryella* and *Collinsella* was significantly greater in the high-dose group (group A2230) compared to the low-dose group (group A30) of dietary AFB1 (*P* < 0.05). The abundance of *Prevotellaceae_NK3B31_group* was significantly greater in groups A445 and A2230 compared to groups A30 and A0. On the other hand, the abundance of *Eubacterium* was significantly lower in the low-dose group compared to the other groups (*P* < 0.05). The control group exhibited considerably larger abundances of *Prevotella_9*, *Agathobacter*, and *Sphingomonadaceae* compared to the AFB1 treated groups (*P* < 0.05).

### 3.7. Muscle Proximate Composition

Table 5 illustrates the proximal composition of the muscle. The muscle moisture content did not exhibit any significant differences between the groups (*P* > 0.05). However, the muscle crude lipid content demonstrated a drop that was dependent on the dosage of dietary AFB1, reaching its lowest value in the A2230 group. On the other hand, the muscle's crude protein content exhibited a consistent upward pattern and reached its highest level in group A2230 (*P* < 0.05). No traces of AFB1 were found in the muscle or liver of group A0. The concentration of AFB1 in the liver exhibited a substantial rise in groups A30, A445, and A2230, with the highest value seen in group A2230 (*P* < 0.05).

### 3.8. Muscle Amino Acids and Fatty Acids Contents

The findings regarding the amino acid composition of the muscle are displayed in Table 6. Glutamic acid (Glu), glycine (Gly), and alanine (Ala) exhibited a positive correlation with the dietary intake of AFB1 among the non-essential amino acids. The levels of isoleucine (Ile), leucine (Leu), valine (Val), phenylalanine (Phe), and lysine (Lys) in groups A445 and A2230 were significantly higher than those in group A0 (*P* < 0.05). Conversely, the levels of arginine (Arg) and threonine (Thr) showed a decreasing trend as the dietary AFB1 increased in this experiment (*P* < 0.05).

The composition of fatty acids in the muscle of the grouper is displayed in Table 7. The amount of saturated fatty acids (SAFA) in group A0 was significantly lower compared to groups A30, A445, and A2230. The highest level of SAFA was found in group A2230 (*P* < 0.05). On the other hand, the total monounsaturated fatty acids (MUFA) increased as the dietary inclusion of AFB1 increased, reaching its highest level in group A2230 (*P* < 0.05). The levels of both polyunsaturated fatty acids (PUFA) and highly unsaturated fatty acids (HUFA) decreased as the amount of dietary AFB1 increased in this experiment. The lowest levels were observed in group A2230 (*P* < 0.05).

## 4. Discussion

### 4.1. Effect of AFB1 on the Growth of Grouper

AFB1 is one of the most toxic aflatoxins and is widely distributed in plant-based raw materials used in the production of diets. As the proportion of plant-based ingredients in grouper diets increases, contamination of diets with AFB1 has become a problem that cannot be ignored [24]. The suppression of aquatic animal growth is a significant harmful consequence of AFB1. The current research has confirmed the findings on Chinese sea bass (*Lateolabrax maculatus*) [25], Nile tilapia (*Oreochromis niloticus*) [26–29], gilthead seabream (*Sparus aurata*) [30], pacific white shrimp (*Litopenaeus vannamei*) [31, 32], and juvenile marbled eel (*Anguilla marmorata*) [33]. The combination of growth and feed conversion ratio indicators with the evaluation of biological characteristics such as body length, carcass weight, fillet weight, CF, CR, and FY provides a comprehensive and reliable assessment of the overall growth and health of the test animals [22]. Elevating the presence of AFB1 in the diet resulted in a decline in WGR, SGR, CF, CR, and FY, while causing a rise in FCR. These findings indicate that AFB1 suppressed the growth of grouper, diminished the proportion of edible portion, and impaired the efficiency of feed consumption. AFB1 was found to reduce WG and SGR in an 85-day feeding experiment with gilthead sea bream [30]. Similarly, in a 56-day study with marbled eel, the final body weight, SGR, and feed efficiency were significantly lower in the AFB1 1,000 *μ*g kg^−1^ of diet group compared to the control group [33]. In addition, in a study with red drum (*Sciaenops ocellatus*), supplementing AFB1 in the diet was found to not only affect growth but also significantly reduce survival [34]. The susceptibility of fish to aflatoxin is contingent upon their growth stage and species. Specifically, juveniles exhibit greater sensitivity compared to adults, and certain species are more susceptible than others [35]. Rainbow trout (*Oncorhynchus mykiss*) exhibit the highest level of sensitivity to AFB1 among all fish species. Long-term exposure to low concentrations of AFB1 (as little as 0.4 *μ*g kg^−1^) has been demonstrated to elevate the likelihood of cancer in rainbow trout [35, 36]. In the present study, there was also no significant difference in the SR of the grouper groups, suggesting that grouper is not sensitive to AFB1. This may be related to the different species of fish used in the experiment. No significant effect of AFB1 on survival was found in Chinese sea bass [25] and gilthead seabream [30]. In addition, tropical fish are more tolerant to AFB1 than cold-water fish [37]. The LD50 of channel catfish (*Ictalurus punctatus*) was determined to be 11.5 mg kg bw^−1^ when administered through intraperitoneal injection, according to a study conducted by Jantrarotai et al. [38]. This LD50 value is roughly 10 times greater than that of rainbow trout. Studies have demonstrated that Nile tilapia has lower susceptibility to AFB1 compared to rainbow trout. Nile tilapia exhibit LD50 values of 1.0 and 1.3 mg kg bw^−1^. However, even after consuming up to 3.0 mg kg^−1^ AFB1, Nile tilapia does not seem to experience any negative effects on their survival [39–42]. Grouper as a warm-water groundfish may also have a higher degree of AFB1 tolerance than cold-water fish.

### 4.2. Effect of AFB1 on the Intestinal Tract of Grouper

The intestine is the main organ of digestion and absorption, and ingestion of AFB1 can trigger oxidative stress in the intestine, leading to impaired intestinal barrier function and thus reducing the growth performance of animals [43].

Histological changes play a crucial role in comprehending the pathogenic effects of diet on fish [44]. The current study found that the consumption of AFB1 in the diet resulted in several histological alterations in the hindgut of hybrid grouper. In the medium dose group, the submucosa became thicker and the width of the villi decreased. In the high-dose group, the integrity of the intestinal tissue was disrupted. The submucosa was invaded by inflammatory cells such as lymphocytes, macrophages, and multicellular cells. The villi became shorter and the number of cup cells decreased. Additionally, the submucosa became thicker and separated from the muscle layer. These findings indicate that diets containing more than 445 *μ*g kg^−1^ AFB1 resulted in structural changes in the intestine. Research conducted on tilapia has demonstrated that diets containing AFB1 can impede fish growth by causing damage to the intestines [45]. Similarly, grass carp showed necrosis, immune cell infiltration, and fibrous hyperplasia in intestinal tissue with increasing AFB1 levels in their diet [46]. In Chinese sea bass, different concentrations of AFB1 led to varying degrees of blurring and damage to the tightly connected structures of the fish intestine, with clear gaps and vacuolation of cells [47]. In a study on rainbow trout, it was found that the presence of AFB1 at 50–100 *μ*g kg^−1^ in the diet caused shortening of the villi and damage to the villi [48]. In this experiment, the expression levels of *jam*, *claudin15*, *claudin3*, and *occludin* were all progressively downregulated with increasing dietary AFB1 concentrations. These findings indicate that the presence of AFB1 in the diet negatively affects the integrity of the tight junctions in the hindgut of the grouper. This aligns with the findings of the histopathological examination of the hindgut. Further research has demonstrated that the increase in pro-inflammatory substances (*tnf-α* and *inf-γ*) typically results in the breakdown of the tight junction barrier in epithelial cells [49]. An increase in pro-inflammatory factors may also affect intestinal permeability, leading to intestinal inflammation [50]. This experiment revealed that *tnf-α* and *inf-γ* in the high-dose AFB1 group were significantly higher than those in the control group, indicating that the high-dose AFB1 group may cause inflammation in the intestine of grouper.

The determination of intestinal antioxidant indicators allows for a better understanding of the mechanism of AFB1′s effects on the intestinal health of hybrid grouper. The results of the intestinal ROS section in this experiment showed a significant increase in intestinal ROS content as the AFB1 content of the diet increased. This indicates that medium to high doses of AFB1 generated a large number of free radicals in the intestine of hybrid grouper. Also in this study, the intestinal MDA content was increased in the high-dose group, indicating that high doses of AFB1 deepen intestinal lipid peroxidation, indirectly responding to an increase in intestinal peroxidative damage. This phenomenon can be ascribed to the metabolic conversion of AFB1 into AFB1-8,9-epoxide, which subsequently interacts with lipids and other macromolecules, resulting in lipid peroxidation and consequent cellular harm [51]. As a member of the stress-related proteins, HSP90 is closely associated with the body's immune protection and can enhance the body's tolerance to a variety of stresses and improve cell viability. In the present experiment, *hsp90* expression levels increased with the increase in diet AFB1 content, suggesting that the intestine may have been exposed to oxidative stress, which corroborates with the results of intestinal ROS.

It is widely understood that the role of antioxidant enzymes in alleviating oxidative stress is crucial, as they scavenge ROS [52]. According to the current study, antioxidant indexes such as SOD, CAT, GPX, and T-AOC were significantly lower in both the medium and high-dose groups compared to the control group. This suggests that the antioxidant capacity of the intestine was reduced by AFB1 in the diet during the 56 days of exposure. Additionally, the intestinal reactive oxygen content increased, leading to dysfunctional intestinal antioxidant function. When exposed to oxidative stress, the production of O_2_ radicals can lead to the oxidation of amino acids and cysteine in enzymes, which results in a decrease in SOD activity [48]. Previous research has shown that in Nile tilapia, dietary AFB1 reduces CAT and SOD activity due to the damaging effect of free radicals (hydrogen peroxide) produced by AFB1-induced oxidative stress [53]. Similarly, in a study with rainbow trout, serum and intestinal MDA content increased with rising diet AFB1, while serum CAT and SOD decreased with increasing feed AFB1 doses [48]. In Chinese sea bass, dietary AFB1 above 1.0 mg kg^−1^ significantly increased MDA content levels and CAT and SOD activities, indicating that these increased antioxidant indicators were in response to physiological toxicity or oxidative stress stimulated by AFB1, rather than an improvement in the antioxidant capacity of fish [25]. Furthermore, a rat study found that feeding 250 *µ*g kg^−1^ body weight day^−1^ for 2 weeks resulted in an increase in liver MDA content and a decrease in GSH activity, which is consistent with the results of this experiment [54]. Organisms have the ability to control the transcription of antioxidant genes through the Keap1–Nrf2 signaling pathway when they are exposed to oxidative stress. The Nrf2 transcription factors are responsible for regulating antioxidant enzymes and have a significant impact on this process [55]. Keap1 functions as a primary inhibitory regulator located upstream of Nrf2, effectively suppressing Nrf2 activity and its associated transcriptional levels [56]. In this study, intestinal *nrf-2* expression levels were consistent with intestinal *sod* and *cat* expression levels, all of which decreased with increasing AFB1 levels, further demonstrating that intestinal antioxidant capacity was inhibited in the mid and high-dose groups.

The activity of digestive enzymes has the ability to affect the utilization of feed and the performance of growth, and it plays a crucial part in the process of digestion [18]. In the present experiment, lipase, amylase, and trypsin all showed decreases in activity with increasing dietary AFB1, suggesting that even low doses (30 *μ*g kg^−1^) of AFB1 in the diet interfered with normal lipid, carbohydrate, and protein absorption in hybrid grouper. The reduced muscle lipid content in the afb1 exposed group in this experiment is most likely because it is already affected at the intestinal digestion and absorption stage, where the reduced lipase activity directly affects the hydrolysis of dietary lipids in the intestine, thus affecting absorption. No significant differences in pancreatic amylase and lipase activity were found in duck studies after ingesting moldy maize (containing AFB1) [57]. In a study on juvenile yellow catfish (*Pelteobagrus fulvidraco*), a significant decrease in amylase and lipase activities was found when the AFB1 concentration in the diet exceeded 100 *μ*g kg^−1^ [43].

The microbiota in our intestines plays a crucial role in maintaining overall health, and dietary components can influence the microbial community and subsequently impact the metabolism and population of important commensal bacteria [20]. *Moryella*, a Gram-positive, non-spore-forming, strictly anaerobic, and non-motile genus of bacteria from the Lachnospiraceae family [58], was found to be significantly more abundant in the high-dose AFB1 group in our experiment. This could be attributed to the immune response in the intestines, as *Moryella* has been found to be linked to *Tfh* and B cell activation during the early stages of the immune response in hosts [59].

In our experiment, we observed a higher abundance of the *Prevotellaceae_NK3B31_group*, a Gram-negative bacterium [60], which produces bacterial endotoxin (Lipopolysaccharide, LPS), in the medium to high-dose AFB1 group. LPS can induce acute inflammation, leading to the secretion of cortisol [61, 62], TNF-*α*, interleukin−1*β* (IL-1*β*), INF-*γ*, and various other inflammatory cytokines and chemokines [63, 64]. Thus, the increase in *Prevotella* in the whole-intestinal microbial community of grouper induced by AFB1 could lead to an elevated production of LPS, resulting in cortisol secretion [65].

*Prevotella_9* is typically associated with a healthy, plant-based diet and is considered a “probiotic” in the human body. Reduced levels of *Prevotella* spp. have been linked to certain diseases, such as autism and behavioral problems in infants [66]. *Prevotella* plays a crucial role in breaking down dietary fiber and generating short-chain fatty acids (SCFAs). In our experiment, the relative abundance of *Prevotella_9* decreased with an increase in AFB1, indicating that AFB1 may impede the synthesis of intestinal SCFAs, consequently affecting lipid metabolism in grouper.

Studies on cattle and sheep have shown that *Prevotella* spp. plays a crucial role in the catabolism of proteins and carbohydrates in feed [67]. SCFAs are byproducts of carbohydrate metabolism, with acetic acid and propionic acid being the predominant SCFAs found in the contents of the colon. The SCFAs can attach to GPR41 and GPR43, which are found in the colon, liver, fat, and skeletal muscle. This attachment helps control the release of glucagon-like peptide-1 (GLP-1) and peptide YY (PYY). The regulation of these hormones can help reduce insulin resistance, leading to an impact on systemic lipid and glucose metabolism [65]. This is evident in our experiment, where an increase in AFB1 caused a decrease in muscle fat content.

### 4.3. Effect of AFB1 on Grouper Muscle

Muscle approximate composition is one of the most intuitive indicators to evaluate the effect of diet on the meat quality of farmed fish [68]. Muscle moisture did not differ significantly between groups in this experiment, muscle crude lipid was significantly lower in the aflatoxin-exposed group than in the control group, while muscle crude protein showed an increasing trend. This suggests that AFB1 in the diet may affect the metabolism of lipids and protein in hybrid grouper. Shrimp (*Penaeus vannamei*) showed a significant decrease in muscle moisture, crude protein, and crude lipid content after 20 days of exposure [69]. After prolonged exposure to high doses of AFB1, one of the essential nutrients of hybrid grouper muscle, crude lipid, was significantly reduced. One of the possible reasons for this is the increased ROS content and reduced antioxidant enzyme activity in hybrid grouper leading to an imbalance in the antioxidant system and redox status of muscle cells, which leads to abnormal oxidative degradation of lipids [70].

Prior investigations have indicated that the presence of AFB1 in the diet can modify the lipid composition of fish or tissues. A study conducted on Nile tilapia demonstrated that the inclusion of 638 *μ*g kg^−1^ AFB1 in the meal had no effect on the lipid content of the muscle, but it did considerably decrease the lipid content in the liver [41]. A separate investigation conducted on Nile tilapia similarly documented that a meal containing AFB1 at a concentration of 3 mg kg^−1^ over a period of 12 weeks resulted in a decrease in the overall lipid content of the entire body [27]. Nevertheless, a study conducted on yellow catfish demonstrated that the overall lipid content of the fish rose when supplemented with 20 and 50 *μ*g kg^−1^ AFB1. Conversely, fish fed with 1,000 *μ*g kg^−1^ AFB1 had a decrease in overall lipid content [71]. The variations in lipid composition across studies can be attributed to disparities in species, AFB1 concentration, and duration of exposure. Moreover, various tissues and the accumulation of lipids throughout the body may elicit distinct physiological reactions when exposed to AFB1 through the food. Nevertheless, additional investigation is necessary. The rise in crude protein may also be due to a decrease in lipid content, resulting in a rise in the relative crude protein content. There are not many studies on AFB1 in aquatic animals. In broiler studies, reduced lipogenic and amino acid metabolizing enzyme activities were found following exposure to AFB1, resulting in reduced lipogenesis [72]. Studies in dairy cattle have reported a major threat to amino acid metabolic pathways under AFB1 exposure [73]. A similar study in goats found that AFB1 exposure altered lipid oxidation, carbohydrate and amino acid metabolism [74].

The liver plays a crucial role in metabolizing, detoxifying, and binding AFB1 to nucleic acids and proteins [75]. Aflatoxin has the ability to accumulate in the liver, muscle, and other edible animal tissues without undergoing any alterations [76]. The current investigation could not find any AFB1 residues in the muscle of the groups. However, residues of 0.23 *μ*g kg^−1^ AFB1 were found in the liver even in the low-dose group, and 1.73 and 2.76 *μ*g kg^−1^ AFB1 in the liver of the medium- and high-dose groups, respectively. This is because the hepatobiliary system is used as an ideal site for the accumulation and excretion of AFB1 and its metabolites [68]. In the matrinxã fish (*Brycon cephalus*) experiment, feeding with a high dose (50 *μ*g kg^−1^ AFB1) for 60 days also revealed the presence of 0.17 *μ*g kg^−1^ AFB1 in the liver, while no AFB1 was detected in muscle [68], and after 180 days, the high-dose group detected 0.17 *μ*g kg^−1^ AFB1 in liver 0.61 *μ*g kg^−1^ and only traces of AFB1 were detected in muscle, indicating that AFB1 residues in tissues were time-dependent.

Essential nutrients such as protein and lipids in fish muscle can be used as important indicators for meat quality evaluation [77]. The composition and content of flavor-presenting amino acids, such as Asp, Glu, Gly, Ala, Phe, and Tyr, affect the flavor of muscle, with Asp, Glu, Gly, and Ala being Umami amino acids (UAA) that enhance the flavor and sweetness of muscle [78]. In this experiment, we found that total amino acid levels in muscle showed an upward trend and Glu, Gly, and Ala were also significantly higher in the high AFB1 dose group than in the control group. However, this did not mean that muscle quality was significantly higher in the AFB1 high-dose group than in the control group; this rise in muscle amino acids was associated with a rise in muscle crude protein and a reduction in crude lipid. In addition to lipid content, the fatty acid composition is also a significant determinant of the nutritional quality of fish. Research has established that reducing intake of SFA and increasing consumption of unsaturated fatty acids, particularly *n*-3 PUFAs, has positive effects on human health [15]. Marine fish often contains significant amounts of *n*-3 PUFA, particularly EPA and DHA, making it widely regarded as a nutritious food for humans [79]. The present experiment revealed that muscle-saturated fatty acids were significantly higher in the AFB1-exposed group than in the control group, while monounsaturated fat, polyunsaturated fatty acids, and highly unsaturated fatty acids showed the opposite trend. This indicates that AFB1 significantly inhibited the accumulation of unsaturated fatty acids in muscle and greatly reduced the nutritional value of grouper fish meat. This may be because AFB1 reduced the lipase activity of grouper in this experiment and damaged the normal structure of the intestine, thus affecting fatty acid absorption. Together with the general perception of liver damage by AFB1 and a corresponding impairment in fat transport, it is speculated that damage to the intestine and liver may be responsible for the reduction in lipid content as well as unsaturated fatty acid content in muscle. In contrast, a study in yellow catfish did not show changes in meat fatty acid composition following dietary AFB1 exposure [80]. This may be due to the different doses of AFB1 in the diets, the AFB1 dose gradient in this experiment being greater than that in the experiment with yellow catfish, and also the possibility that yellow catfish is more tolerant of AFB1 exposure than grouper.

It has been shown that there is a strong correlation between muscle fiber characteristics and the textural properties of the muscle, with the smaller the muscle fiber diameter and the higher the density of muscle fibers, the better the firmness of the muscle, improving stiffness and chewiness and thus taste [81–83]. Muscle fiber characteristics are intricately linked to muscle growth, which can be categorized into two main processes: hyperplasia and hypertrophy. Hyperplasia refers to the increase in the number of muscle fibers during muscle growth, while hypertrophy involves the enlargement of the diameter of muscle fibers. These two processes, hyperplasia and hypertrophy, work in tandem to manifest muscle growth [82]. The growth of animals is mainly the growth of skeletal muscle.

The formation of myofibrils is controlled by several factors, such as myogenic regulatory factors (MRFs) and myocyte enhancer factor 2 (*mef2*), which have a positive effect, and myostatin (*mstn*), which has a negative effect [84].

The study has found four members of the MRFs family, specifically *myod*, *myf5*, *myog*, and *mrf4*. *Myod* and *myf5* are primarily expressed during the initial phases of myogenesis and play a role in generating and sustaining myogenic cells. On the other hand, *myog* and *mrf4* primarily participate in the final differentiation of myoblasts and act as the primary regulators of gene expression for skeletal muscle-specific proteins [84, 85]. In this experiment, the medium to a high dose of AFB1 significantly inhibited the expression levels of muscle *myog* and *mrf4* and upregulated muscle *mstn*, while *myod* and *myf5* did not differ significantly between the groups. These findings indicate that increased levels of AFB1 in the diet hinder the process of myoblast differentiation and growth, as well as decrease the division and proliferation of mature myoblasts, leading to reduced muscle development [86]. However, the presence of AFBI in the diet does not appear to have an impact on myoblast production. Collagen type I *α*1 (*col1a-1*) and collagen type I *α*2 (*col1a-2*) govern the dimensions and rigidity of muscle fibers [87]. In the present experiment, muscle *col1a-1* and *col1a-2* expression levels were significantly higher in the control and AFB1 low-dose groups than in the medium and high-dose groups. This suggests that muscle fiber diameter and stiffness are inhibited at medium to high doses of AFB1 exposure, as evidenced by the results of our muscle histological sections, where muscle gaps became larger and muscle diameters became shorter in the medium to high-dose group. In addition, these changes may be related to the oxidative stress damage to the muscle. The investigation showed a substantial decrease in *sod* expression levels in the group exposed to AFB1 compared to the control group. Moreover, the expression levels of *keap1* were found to rise proportionally with the intake of AFB1, while the expression levels of *nrf-2* were repressed. This indicates that the muscle of the grouper may have suffered damage due to oxidative stress caused by exposure to AFB1. In a study on Nile tilapia, AFB1 exposure was also found to significantly reduce the relative area of myofibrils [80]. Another study in shrimp and tilapia also found that AFB1 exposure caused similar muscle damage in shrimp and fish, including damage to myogenic fibers and a larger gap between myofilaments, but the extent of damage was more pronounced in shrimp muscle than in fish [32].

A further investigation on shrimp proposed that the harm inflicted on shrimp muscle by AFB1 might be attributed to oxidative stress induced by substantial concentrations of AFB1, as well as the suppression of protein synthesis leading to DNA damage and death [69]. Currently, our understanding of the processes via which AFB1 hinders muscle fiber development in fish is limited, and additional investigation is required.

## 5. Conclusion

In summary, diets containing more than 30 *μ*g kg^−1^ AFB1 inhibited the growth of hybrid grouper. When AFB1 levels in the diet exceeded 445 *μ*g kg^−1^, hybrid grouper showed increased hindgut ROS levels, increased inflammatory response, increased intestinal permeability, decreased hindgut structural integrity, and reduced digestive enzyme activity. The presence of AFB1 in the experiment led to a decrease in beneficial intestinal bacteria, such as *Prevotella*, and an increase in harmful intestinal bacteria, such as *Prevotellaceae_NK3B31_group*. Muscle-related results indicated that AFB1 in the diet reduced muscle lipid content, increased saturated fatty acid content in muscle, decreased unsaturated fatty acid levels in muscle, increased muscle interstitial space, and reduced muscle fiber diameter, thereby deteriorating grouper muscle quality.

## Figures and Tables

**Table 1:** Ingredient composition and nutrient content of experimental diets (% dry matter).

Ingredients	A0	A30	A445	A2230
Brown fish meal	43	43	43	43
Soybean protein concentrated	13	13	13	13
Wheat gluten	6	6	6	6
Chicken meal	11	11	11	11
Wheat flour	15	15	15	15
Soybean lecithin	1.5	1.5	1.5	1.5
Fish oil	3	3	3	3
Calcium dihydrogen phosphate	1	1	1	1
Choline chlorine	0.5	0.5	0.5	0.5
Vitamin and Mineral Premix^a^	1	1	1	1
Microcrystalline cellulose	4.82	4.82	4.82	4.82
Antioxidant	0.03	0.03	0.03	0.03
Attractante	0.15	0.15	0.15	0.15
Supplemented AFB1 (*μ*g kg^−1^)	0	50	500	2,500
Proximate composition				
Dry matter	92.23	91.62	92.13	92.17
Crude protein	49.03	49.24	49.67	49.10
Crude lipid	9.20	9.28	9.09	9.24
Determined AFB1 (*μ*g kg^−1^)	0.00	30.40	445.00	2230.00

^a^Vitamin and Mineral Premix (kg^−1^ of diet): thiamin, 5 mg; riboflavin, 10 mg; vitamin A, 5,000 IU; vitamin E, 40 mg; vitamin D3, 1,000 IU; menadione, 10 mg; pyridoxine, 10 mg; biotin, 0.1 mg; cyanocobalamin, 0.02 mg; calcium pantothenate, 20 mg; folic acid, 1 mg; niacin, 40 mg; vitamin C, 150 mg; iron, 100 mg; iodine, 0.8 mg; cupper, 3 mg; zinc, 50 mg; manganese, 12 mg; selenium, 0.3 mg; cobalt, 0.2 mg.

**Table 2:** Sequences of the primer pairs used for real-time quantitative RT-PCR.

Gene	5 = /3 = Forward primer	5 = /3 = Reverse primer	Amplicon	Genbank no.
*jam*	GTCTGCCTGGTCATTGTG	ACCTTCCTCTGTGTCCTG	85	XM_033615938.1
*claudin15*	GGTTACATCCAAGCGTCTCG	TGCCAGCAATCCTCCCTTT	139	XM_033651910.1
*claudin3*	TGAGACACAGAAGAGGGAGATAG	CCCTCCAATGAGCAGAAATGA	76	XM_033630592.1
*zo-1*	CCAGCAGCCATACAGAGATTAC	GGCGGGTTAGAGTCATAGTTTC	120	XM_033628716.1
*occludin*	CTGTCACTGTCTATAAGCTACGCTC	TCTTAACACTTTGCACATGAAGTGGA	109	XM_033622284.1
*tnf-α*	GCAGCGATGGTGACGAGAAGG	TCCTCCTGTGCCGTGCTCTG	142	XM_033640148.1
*nrf-2*	ACTGATCTGCCGTTCTCTTTC	TTTCTGACGGTGGTTGTAGTC	111	XM_033617941.1
*keap1*	TCCACAAACCCACCAAAGTAA	TCCACCAACAGCGTAGAAAAG	209	XM_033623805.1
*inf-γ*	CGATTCGGTCATCAAGAGCAT	CTCCGTCACGACCGACACCA	136	XM_033624581.1
*hsp90*	AACGACAAGGCTGTGAAGGAC	TTCTGTAGATGCGGTTGGAGTG	109	XM_033648354.1
*sod*	CAGTGGGACCGTGTATTTTGAG	CAGTCACATTTCCCAGGTCTCC	224	XM_033633905.1
*cat*	TCGGCAAGACTACACCTA	GAGAGTGGATGAAGGATGG	195	XM_033635388.1
*myog*	TTATCCCGTGGTCCAGAGGT	GGTGTCGGGTTCATGCAGTA	88	XM_033625713.1
*col1a1*	CTAAGGGAGAGGCTGGAGATAA	CTTAGGGCCAGTGTTTCCAA	113	XM_033645844.1
*col1a2*	CCACTATCAAGTCCCTCAACAC	GTAGAATCCGCTGCTCCATT	125	XM_033636413.1
*mrf4*	AGACGCTGGACGAGCAGGAGAA	AGTGGAATGGTCGGCAGAGGT	125	XM_033614362.1
*myf5*	ACGAGAGCAGGTGGAAAACTA	TTATCGCCTAAACTCTCGTTCT	177	XM_033614452.1
*mstn*	CTTTGGCTGGGACTGGATTAT	CCTCTGGGATTGGCTTTGT	126	XM_033614507.1
*myod*	CTGAAAGTGTGGAGGCTCGT	GATGAACACTGTGCGAAGCG	153	XM_033635008.1
*4ebp1*	TGTCAACTGAGTGCCAGAAG	CTCCGGGAGTAGTGGAGTAG	108	XM_033618653.1
*mtor*	CAGGTGGCTAGTACAGGTTATG	CTCTCTTCTGATGCCCTGATTT	111	XM_033629179.1
*β-actin*	GGCTACTCCTTCACCACCACA	TCTGGGCAACGGAACCTCT	188	XM_033645256.1

*Note*: *jam*, junctional adhesion molecule 1; *zo-1*: tight junction proteins ZO-1; *tnf-α*, tumor necrosis factor-*α*; *nrf-2*, nuclear factor erythroid 2-related factor 2; *keap1*, kelch-like ECH-associated protein 1; *inf-γ*, interferon-*γ; hsp90*, heat shock protein 90; *sod*, superoxide dismutase; *cat*, catalase; *myog*, myogenin; *col1a1*, collagen *α*-1(I) chain; *col1a2*, collagen *α*-2(I) chain; *mrf4*, myogenic factor 4; *myf5*, myogenic factor 5; *mstn*, myostatin; *myod*, myogenic differentiation antigen; *4ebp1*, eukaryotic translation initiation factor 4E binding protein 1; *mtor*, mammalian target of rapamycin.

**Table 3:** Growth and feed utilization of hybrid grouper fed the experimental diets containing different AFB1 levels for 8 weeks.

Item	A0	A30	A445	A2230
IBW (g)	11.60 ± 0.01	11.61 ± 0.02	11.60 ± 0.02	11.61 ± 0.01
SR (%)	90.00 ± 5.77	95.56 ± 1.92	94.44 ± 1.92	95.56 ± 1.92
WGR (%)	748.81 ± 20.32^d^	677.63 ± 3.00^c^	605.33 ± 13.14^b^	360.91 ± 8.22^a^
SGR (% day^−1^)	3.45 ± 0.11^c^	3.36 ± 0.03^c^	3.14 ± 0.03^b^	2.26 ± 0.06^a^
FCR	0.87 ± 0.04^a^	0.90 ± 0.02^a^	0.99 ± 0.05^a^	1.54 ± 0.06^b^
CF (%)	658.65 ± 30.89^d^	610.56 ± 22.18^cd^	506.05 ± 115.46^b^	388.41 ± 34.91^a^
CR (%)	69.63 ± 3.26^ab^	73.88 + 7.88^b^	58.03 ± 3.94^a^	59.46 ± 3.34^a^
FY (%)	52.58 ± 1.54^c^	49.00 + 3.96^bc^	44.21 ± 0.89^ab^	40.23 ± 3.51^a^

*Note*: Values in the table are means ± SEM (*n* = 3); values in the same row with different superscripts indicate significant differences (*P* < 0.05). Initial mean body weight (IBW, g per fish). Survival rate (SR, %) = 100 × final fish number/initial fish number. Weight gain rate (WGR, %) = 100 × (final body weight (g)−initial body weight (g))/initial body weight (g). Specific growth rate (SGR, %) = 100 × (ln final body weight (g)–ln initial body weight (g))/days of experiment. Feed conversion ratio (FCR) = feed intake (g)/(final body weight (g)–initial body weight (g)). Condition factor (CF, %) = 100 × body wet weight (g)/body length (cm)^3^. Carcass ratio (CR, %) = 100 × Carcass weight/Body weight (where carcass is the edible part of the fish body, removed of head, viscera, fins and fish tail). Filet yield (FY, %) = 100 × fillets weight/Body weight (where fillets are the edible part of the fish, deboned and skinless).

**Table 4:** Whole-intestinal enzyme activity and antioxidant parameters of hybrid grouper fed the experimental diets containing different AFB1 levels for 8 weeks.

Item	A0	A30	A445	A2230
MDA	621.1 ± 28.38^a^	832.23 ± 37.62^ab^	730.91 ± 169.77^ab^	876.42 ± 24.51^b^
SOD	280.46 ± 14.21^b^	179.83 ± 39.72^a^	164.3 ± 15.44^a^	146.87 ± 15.10^a^
CAT	72.31 ± 1.57^c^	56.17 ± 1.53^b^	60.97 ± 4.28^b^	31.65 ± 2.72^a^
GPx	244.96 ± 18.62^c^	163.75 ± 18.96^b^	138.13 ± 13.94^ab^	125.68 ± 8.06^a^
ROS	419.85 ± 34.34^a^	451.69 ± 24.56^ab^	521.31 ± 38.71^c^	499.99 ± 12.45^bc^
T-AOC	0.32 ± 0.03^c^	0.23 ± 0.02^b^	0.17 ± 0.01^a^	0.16 ± 0.01^a^
LIP	1,034.72 ± 42.78^b^	744.74 ± 71.18^a^	692.7 ± 65.16^a^	606.6 ± 131.24^a^
AMS	594.35 ± 65.85^c^	481.63 ± 18.82^b^	315.89 ± 24.63^a^	307.14 ± 26.08^a^
TRY	5,515.83 ± 186.67^b^	3,723.25 ± 274.61^a^	3,295.81 ± 545.06^a^	3,032.13 ± 40,751^a^

*Note*: Values in the table are means ± SEM (*n* = 3); values in the same row with different superscripts indicate significant differences (*P* < 0.05). MDA (nmol mg^−1^·pro): malondialdehyde, SOD (U mg^−1^·pro): superoxide dismutase, CAT (U mg^−1^·pro): catalase, GPx (mU mg^−1^·pro): glutathione peroxidase, ROS (fluorescence intensity mg^−1^·pro): reactive oxygen species, T-AOC (*μ*mol Trolox mg^−1^·pro): total anti-oxidation capacity, LIP (mU mg^−1^·pro): Lipase, AMS (mIU mg^−1^·pro): *α*-amylase, TRY (U mg^−1^·pro): Trypsin.

**Table 5:** Muscle proximate composition and AFB1 content in tissues of hybrid grouper fed the experimental diets containing different AFB1 levels for 8 weeks (wet basis).

Item	A0	A30	A445	A2230
Moisture (%)	77.25 ± 0.11	76.45 ± 0.49	76.49 ± 0.40	76.52 ± 0.26
Crude lipid (%)	1.84 ± 0.11^c^	1.47 ± 0.10^b^	1.47 ± 0.05^b^	1.14 ± 0.13^a^
Crude protein (%)	19.04 ± 0.30^a^	19.87 ± 0.35^b^	20.45 ± 0.02^bc^	20.84 ± 0.14^c^
Muscle AFB1 (*μ*g kg^−1^, dry matter base)	—	—	—	—
Liver AFB1 (*μ*g kg^−1^, dry matter base)	—	0.23 ± 0.06^a^	1.73 ± 0.71^b^	2.76 ± 0.08^c^

*Note*: Values in the table are means ± SEM (*n* = 3); values in the same row with different superscripts indicate significant differences (*P* < 0.05). Crude lipid, crude protein, muscle AFB1, and liver AFB1 were assayed on a dry matter basis.

**Table 6:** Muscle amino acids contents of hybrid grouper fed the experimental diets containing different AFB1 levels for 8 weeks.

Item	A0	A30	A445	A2230
NEAA^a^				
Asp	9.07 ± 0.04^a^	9.13 ± 0.01^a^	9.36 ± 0.04^b^	9.10 ± 0.05^a^
Tyr	2.83 ± 0.02	2.82 ± 0.04	2.89 ± 0.08	2.93 ± 0.03
Ser	3.41 ± 0.03	3.53 ± 0.04	3.49 ± 0.07	3.41 ± 0.06
Glu	12.77 ± 0.19^a^	13.19 ± 0.07^b^	13.29 ± 0.07^b^	13.24 ± 0.08^b^
Gly	4.66 ± 0.05^a^	4.76 ± 0.04^a^	4.70 ± 0.08^b^	5.02 ± 0.08^b^
Ala	5.00 ± 0.09^a^	5.28 ± 0.05^b^	5.25 ± 0.04^b^	5.39 ± 0.07^b^
Pro	3.33 ± 0.10	3.39 ± 0.07	3.28 ± 0.05	3.56 ± 0.17
EAA^b^				
Met	2.50 ± 0.05	2.60 ± 0.03	2.51 ± 0.06	2.59 ± 0.12
Ile	3.64 ± 0.03^a^	3.94 ± 0.04^b^	3.95 ± 0.04^b^	3.96 ± 0.02^b^
Leu	6.64 ± 0.03^a^	6.82 ± 0.02^b^	6.85 ± 0.04^c^	6.90 ± 0.02^c^
Val	3.99 ± 0.03^a^	4.07 ± 0.02^ab^	4.00 ± 0.07^a^	4.16 ± 0.05^b^
Phe	3.26 ± 0.05^a^	3.45 ± 0.04^b^	3.63 ± 0.04^c^	3.71 ± 0.02^c^
His	1.92 ± 0.05	2.00 ± 0.08	2.05 ± 0.06	1.96 ± 0.05
Lys	7.94 ± 0.11^a^	8.19 ± 0.06^b^	8.24 ± 0.03^b^	8.22 ± 0.01^b^
Arg	5.06 ± 0.05^ab^	5.16 ± 0.05^bc^	5.23 ± 0.03^c^	4.96 ± 0.08^a^
Thr	3.94 ± 0.04^ab^	4.03 ± 0.02^b^	4.02 ± 0.05^ab^	3.87 ± 0.10^a^
TAA^c^	79.98 ± 0.41^a^	82.81 ± 0.47^b^	82.75 ± 0.38^b^	82.98 ± 0.13^b^

*Note*: Values in the table are means ± SEM (*n* = 3); values in the same row with different superscripts indicate significant differences (*P* < 0.05). ^a^NEAA, nonessential amino acid; ^b^EAA, essential amino acid; ^c^TAA, total amino acids.

**Table 7:** Muscle fatty acids contents of hybrid grouper fed the experimental diets containing different AFB1 levels for 8 weeks.

Item	A0	A30	A445	A2230
C14 : 0	2.86 ± 0.02^a^	3.78 ± 0.22^c^	4.03 ± 0.02^c^	3.46 ± 0.01^b^
C15 : 0	0.33 ± 0.02^a^	0.45 ± 0.01^b^	0.51 ± 0.01^c^	0.61 ± 0.02^d^
C16 : 0	24.08 ± 0.01^a^	32.34 ± 0.06^b^	35.41 ± 0.06^c^	32.65 ± 0.01^b^
C17 : 0	0.69 ± 0.02^a^	0.81 ± 0.06^b^	0.88 ± 0.04^b^	1.08 ± 0.01^c^
C18 : 0	7.96 ± 0.02^a^	10.54 ± 0.04^b^	12.22 ± 0.03^bc^	13.42 ± 0.05^c^
C20 : 0	0.59 ± 0.02^a^	0.72 ± 0.02^b^	0.83 ± 0.06^b^	0.91 ± 0.03^c^
C22 : 0	0.32 ± 0.01^a^	0.53 ± 0.11^a^	1.00 ± 0.07^b^	1.23 ± 0.01^b^
C24 : 0	0.12 ± 0.02^a^	0.18 ± 0.05^ab^	0.25 ± 0.05^b^	0.30 ± 0.03^c^
∑SAFA^a^	33.75 ± 0.03^a^	45.09 ± 0.17^b^	50.59 ± 0.04^c^	49.58 ± 0.04^c^
C15 : 1n7	3.41 ± 0.06	3.47 ± 0.08	3.42 ± 0.10	3.41 ± 0.03
C16 : 1n7	3.91 ± 0.01^a^	5.09 ± 0.01^c^	4.95 ± 0.02^c^	4.56 ± 0.05^b^
C17 : 1n7	0.28 ± 0.03	0.21 ± 0.01	0.19 ± 0.01	0.28 ± 0.03
C18 : 1n9t	0.19 ± 0.03^a^	0.25 ± 0.02^b^	0.28 ± 0.03^b^	0.29 ± 0.02^c^
C18 : 1n9c	22.7 ± 0.03^a^	28.21 ± 0.09^b^	29.72 ± 0.05^b^	30.36 ± 0.02^c^
C20 : 1n9	1.91 ± 0.02^a^	2.22 ± 0.03^b^	2.35 ± 0.02^c^	2.35 ± 0.05^c^
C22 : 1n9	0.35 ± 0.01^a^	0.41 ± 0.01^ab^	0.46 ± 0.01^b^	0.53 ± 0.04^c^
C24 : 1n9	0.47 ± 0.03^a^	0.54 ± 0.03^ab^	0.66 ± 0.02^bc^	0.79 ± 0.05^c^
∑MUFA^b^	29.80 ± 0.03^a^	36.92 ± 0.12^b^	38.59 ± 0.08^c^	39.15 ± 0.09^c^
C18 : 2n6t	0.17 ± 0.02	0.18 ± 0.03	0.19 ± 0.05	0.21 ± 0.02
C18 : 2n6c	12.1 ± 0.03^c^	8.21 ± 0.04^b^	4.81 ± 0.02^a^	5.83 ± 0.05^a^
C18 : 3n3	1.19 ± 0.04^c^	0.56 ± 0.02^b^	0.19 ± 0.01^a^	0.20 ± 0.03^a^
C20 : 2	0.63 ± 0.05^c^	0.37 ± 0.02^b^	0.20 ± 0.05^a^	0.24 ± 0.02^a^
C20 : 3n6	0.26 ± 0.05^c^	0.14 ± 0.01^b^	0.00 ± 0.00^a^	0.00 ± 0.00^a^
C20 : 3n3	0.15 ± 0.01^b^	0.00 ± 0.00^a^	0.00 ± 0.00^a^	0.00 ± 0.00^a^
C20 : 4n6	1.02 ± 0.02^c^	0.36 ± 0.04^b^	0.00 ± 0.00^a^	0.00 ± 0.00^a^
C20 : 5n3	8.04 ± 0.08^c^	1.97 ± 0.06^b^	0.41 ± 0.05^a^	0.37 ± 0.04^a^
C22 : 6n3	9.46 ± 0.06^c^	1.87 ± 0.02^b^	0.37 ± 0.01^a^	0.37 ± 0.03^a^
∑HUFA^c^	17.51 ± 0.05^c^	3.83 ± 0.03^b^	0.78 ± 0.04^a^	0.76 ± 0.04^a^
∑PUFA^d^	32.53 ± 0.62^c^	13.64 ± 0.08^b^	6.16 ± 0.06^a^	7.22 ± 0.03^a^

*Note*: Values in the table are means ± SEM (*n* = 3); values in the same row with different superscripts indicate significant differences (*P* < 0.05). ^a^∑SAFA, Total saturated fatty acid; ^b^∑MUFA, Total monounsaturated fatty acids; ^c^∑HUFA, 20 : 5n−3 and 22 : 6n−3; ^d^∑PUFA, Total polyunsaturated fatty acids.

## Data Availability

The data that support the findings of this study are available on request from the corresponding author. The data are not publicly available due to privacy or ethical.

## Supplementary Materials

Supplementary Materials Reporting checklist for study using laboratory animals.
